# Expression of B7-H6 expression in human hepatocellular carcinoma and its clinical significance

**DOI:** 10.1186/s12935-018-0627-7

**Published:** 2018-09-04

**Authors:** Lujun Chen, Jun Feng, Bin Xu, You Zhou, Xiao Zheng, Changping Wu, Jingting Jiang

**Affiliations:** 1 Department of Tumor Biological Treatment and Research Center for Cancer Immunotherapy Technology of Jiangsu Province, The Third Affiliated Hospital of Soochow University, Changzhou, 213003 Jiangsu China; 2 Jiangsu Engineering Research Center for Tumor Immunotherapy, The Third Affiliated Hospital of Soochow University, Changzhou, 213003 Jiangsu China; 3 Institute of Cell Therapy, The Third Affiliated Hospital of Soochow University, Changzhou, 213003 Jiangsu China

**Keywords:** B7-H6, HCC, Immunohistochemistry, RNAi, Cancer progression

## Abstract

**Background:**

Recent studies have suggested that B7-H6, a new member of the B7 family of ligands, not only is a crucial regulator of NK cell-mediated immune responses but also has important clinical implications due to its abnormal expression in many human cancers. We have previously reported that higher B7-H6 expression levels in ovarian cancer tissues are positively correlated with tumor metastasis and cancer progression. To date, the expression of B7-H6 in human hepatocellular carcinoma (HCC) and the clinical significance of B7-H6 expression still remain elusive.

**Methods:**

In the present study, the expression level of B7-H6 was examined in both HCC tissues and HCC cell lines (HepG2 and SMMC-7721). And the clinical significance of B7-H6 was analyzed as well.

**Results:**

Our results revealed that B7-H6 was expressed abnormally in HCC tissues, which was greatly related to tumor size. The TCGA data also showed that the B7-H6 mRNA expression level was significantly negatively correlated with the survival of HCC patients. Next, to investigate the functions of B7-H6 in HCC, we successfully constructed B7-H6 knockdown expression human HCC cell lines using the RNA interference technology. Our studies showed that reduced expression of B7-H6 in HepG2 and SMMC-7721 cells significantly attenuated cell proliferation as well as cell migration and invasion. Besides, depletion of B7-H6 greatly induced cell cycle arrest at G1 phase. And also B7-H6 knockdown in HCC cell lines dramatically decreased the C-myc, C-fos and Cyclin-D1 expression.

**Conclusions:**

Our present findings suggested that B7-H6 played an important role in oncogenesis of HCC on cellular level, and B7-H6 could be employed to develop immunotherapeutic approaches targeting this malignancy.

## Background

B7-H6, also known as NCR3LG1 or DKFZp686O24166, is a newly identified ligand in the B7 family [1, 2]. B7-H6 is a type I transmembrane protein that shows considerable homology with the B7-H1 and B7-H3 proteins [3]. B7-H6 binds to its activating receptor, NKp30, on natural killer (NK) cells and then initiates innate immune responses for cellular transformation [1, 2]. To date, numerous studies have demonstrated that although B7-H6 is not expressed in normal human tissues, its expression can be detected in many types of human tumor cells and that the overexpression of B7-H6 in human cancer tissues is significantly associated with prognoses and other clinicopathological parameters of patients, thus suggesting that B7-H6 can serve as a potential tumor marker [1, 2, 4–7]. In addition, our previous study reported that B7-H6 is highly expressed in human ovarian cancer tissues and that its expression level is significantly associated with cancer progression and patients’ prognoses [8].

As one of the most common cancers worldwide, hepatocellular carcinoma (HCC) has been shown to be one of the leading causes of cancer-related deaths, especially in China [9]. Hepatitis B virus (HBV) infection is a major cause of HCC [10], and NKp30-B7-H6 interaction could aggravate hepatocyte damage through the upregulation of interleukin-32 in HBV-related acute-on-chronic liver failure [11]. However, to date, the exact role of B7-H6 expression in the oncogenesis and progression of HCC remains largely unexplored. In the present study, we aimed to identify the clinical significance of B7-H6 expression in human HCC tissues and to further investigate the role of B7-H6 expression in regulating cellular functions in HCC cell lines.

## Materials and methods

### Patients and sample collection

The HCC tissue array HLiv-HCC180Sur-05 was purchased from Shanghai Outdo Biotech Co., Ltd. (Shanghai, P. R. China). A total of 90 HCC patients (from 25 to 73 years of age, with a median age of 56) who underwent surgery between February 2007 and March 2009 were enrolled in the present study. No patient received preoperative chemotherapy or radiotherapy, and all tumor tissues were confirmed as HCC using hematoxylin and eosin (H&E) staining after surgical resection. Incomplete tissue samples and several missing tissue samples were excluded during the heat-induced antigen retrieval. A final total of 87 cases were included in the statistical analysis. Table 1 presents the detailed clinical parameters of the patients.

**Table 1:** Correlation between the B7-H6 expression level in HCC tissues and the patients’ clinical parameters

Clinical parameters	Cases	B7-H6 expression level			*P*
		*H*-*score *≤ 65	*H*-*score *>65	*χ* ^2^	
Gender				0.464	0.496
Male	78	18	60		
Female	9	3	6		
Age (years)				5.943	*0.015*
< 55	45	6	39		
≥ 55	42	15	27		
Tumor size (cm)				4.519	*0.034*
≤ 5	58	18	40		
> 5	29	3	26		
Pathological stage				2.114	0.146
I + II	57	11	46		
III	30	10	20		
Pathological type				3.104	0.078
Massive	52	16	36		
Nodular	35	5	30		
T stage				0.036	0.849
I + II	43	10	33		
III + IV	44	11	33		

Italic font signifies *P *< 0.05

### Antibodies and major reagents

The rabbit anti-human B7-H6 polyclonal antibody was purchased from Abcam (Cambridge, MA, USA). The HRP-conjugated goat anti-mouse/rabbit secondary antibody (K500711, ready to use) was obtained from Dako (Glostrup, Denmark). A rabbit anti-human GAPDH antibody (Sigma, St. Louis, MO, USA) was used for Western blot analysis. The RNeasy Mini Kit was supplied by Qiagen (Valencia, CA, USA), and SYBR Green Master Mix kits were provided by TaKaRa (Dalian, China). DMEM and fetal bovine serum (FBS) were purchased from Gibco (Cambrex, MD, USA).

### Immunohistochemistry

Immunohistochemical staining was performed as described in our previous reports [12–15]. Briefly, the HCC tissue array block was cut into 4-µm-thick sections, and the sections were dewaxed in xylene and rehydrated in a graded series of alcohols. Antigens were retrieved by heating the tissue sections at 100 °C for 30 min in an EDTA (1 mM, pH 9.0) solution. Cooled tissue sections were immersed in 0.3% hydrogen peroxide solution for 15 min to block endogenous peroxidase activity, rinsed with phosphate-buffered saline (PBS) for 5 min and blocked with a 3% BSA solution at room temperature for 30 min. Next, the sections were incubated with a rabbit polyclonal antibody against human B7-H6 (1:150) at 4 °C overnight and were then incubated with an HRP-conjugated goat anti-rabbit secondary antibody. Diaminobenzene was used as the chromogen, and hematoxylin was used as the nuclear counterstain. Finally, the sections were dehydrated, cleared and mounted.

### Evaluation of immunohistochemical staining

*H*-*score* was used to assess the immunostaining intensity of B7-H6 [14, 16], which was calculated as follows: $$\begin{aligned} H \, score\, = \, & \left( {\% {\text{ tumor cells unstained}}\, \times \,0} \right)\, + \,\left( {\% {\text{ tumor cells stained weak}}\, \times \, 1} \right) \\ & + \,\left( {\% {\text{ tumor cells stained moderate}}\, \times \, 2} \right)\, + \,\left( {\% {\text{ tumor cells stained strong}}\, \times \, 3} \right) \\ \end{aligned}$$, ranging from 0 (100% negative tumor cells) to 300 (100% strongly stained tumor cells). Two independent senior pathologists examined all HCC tissue sections blindly. The averaged score was calculated and further used for statistical analysis.

### shRNA

RNA interference technology was used to establish stable cell lines. Small hairpin RNA (shRNA) against human B7-H6 gene (NM_001202439.2; GenBank) was obtained from Shanghai Generay Biotech Co., Ltd. (Shanghai, China). The shRNA target sequence against B7-H6 was as follows: 5′-CATCAAGAATATGGATGGCACATTT-3′. And the non-targeted control sequence was 5′-TTCTCCCCGAACAACAACGTGTCACCACCACGT-3′. shRNA was cloned into a lentiviral vector encoding green fluorescent protein (GFP).

### Cell culture and treatments

Human HCC cell lines HepG2 and SMMC-7721 were obtained from Chinese Academy of Sciences, Shanghai Institutes for Biological Sciences, which were cultured in standard DMEM supplemented with 10% fetal bovine serum under standard culture conditions (5% CO_2_, 37 °C). To establish stable B7-H6 knockdown HCC cell lines, lentivirus was used. The recombinant B7-H6-targeting lentivirus (LV-B7-H6-shRNA virus) or control mock lentivirus (LV-NC virus) were transfected into HepG2 and SMMC-7721 cells. Using an Aria II flow sorter (BD Bioscience, NJ, USA), the GFP-positive cells were subsequently sorted from the two stable cell lines, which were then respectively named as LV-B7-H6-shRNA or LV-NC.

### RNA isolation and real-time PCR (RT-PCR)

RT-PCR was performed to confirm the knockdown of B7-H6 expression at the mRNA level in HepG2 and SMMC-7721 cells. Total RNA was extracted using TRIzol (Invitrogen) reagent, and purified RNA was then reversely transcribed to cDNA using an RT reaction kit (Promega). RT-PCR was conducted using the ABI 7600 system (Applied Biosystems, USA), and SYBR Green was used as a DNA-specific fluorescent dye. Human GAPDH was selected as a housekeeping gene. Primers were synthesized as follows: GAPDH forward: 5′-TGACTTCAACAGCGACACCCA-3′ and GAPDH reverse: 5′-CACCCTGTTGCTGTAGCCAAA-3′; and human B7-H6 forward: 5′-CTCCTGATTCTGCTGTGGGC-3′ and human B7-H6 reverse: 5′-GTCGGAATGCCTCTTGGTGA-3′. In addition, RT-PCR products were confirmed by electrophoresis on a 1.8% agarose gel containing 0.1% ethidium bromide. Images of the fluorescent bands were captured using a Bio-Rad gel documentation system.

### Cell proliferation assay

The cell proliferation was performed using Cell Counting Kit-8 (CCK-8, Beyotime, Shanghai, China) according to the manufacturer’s instructions. 3 × 10^4^ cells were seeded into 96-well plates and maintained in 100 μL of standard DMEM supplemented with 10% FBS. Then culture medium was replaced with CCK-8 reagent (10 μL CCK-8) at indicated time points. After incubating for 1 h, the absorbance of each well was examined at a wavelength of 450 nm. The relative absorbance of experimental values to the initial values indicates cell growth or death, respectively. Cell growth was monitored every 24 h over a period of 3 days. Each experiment was repeated for at least three times.

### Wound healing assay

Cell migration ability was assessed using a wound scrape assay to examine the role of B7-H6 in regulating the migration ability of HCC cell lines, as described in our previous studies [12, 17, 18]. Briefly, cells from the LV-B7-H6-shRNA and LV-NC groups were maintained in 6-well plates. A small wound area was created using a 200-µL pipette tip when cells reached a confluence of 90%. Cells were then washed twice with PBS and were further incubated in serum-free DMEM at 37 °C in a 5% CO_2_ incubator for 0 h, 12 h, 24 h and 36 h. Images were acquired at the indicated time points, and the wound width was measured using a BX50 microscope (Olympus^®^) at 100× magnification with a calibrated eyepiece grid (1 mm/20 μm graduations). A total of 10 measurements were made at random intervals along the wound length, and data from three independent experiments were averaged and expressed as a percentage of the original width. Each experiment was conducted in triplicate.

### Transwell invasion assay

The Transwell culture system was used to examine the invasive ability of the HCC cell lines after knockdown of B7-H6 expression, as described in our previous studies [12, 17, 18]. Briefly, the upper chamber of Transwell^®^ inserts with a pore size of 8 μm and a diameter of 6.5 mm was coated with 20 μL of Matrigel (diluted 1:3 in serum-free DMEM) and was incubated at 37 °C for 4 h. Cells from the LV-B7-H6-shRNA and LV-NC groups were harvested by trypsinization after 12 h of serum starvation and were then resuspended in a serum-free DMEM at a density of 1 × 10^6^ cells/mL. Subsequently, 200 μL of the cell suspension was added to the upper chamber of the coated inserts, while 600 μL of DMEM supplemented with 10% FBS was placed in the bottom chamber as a chemoattractant. After incubation at 37 °C for 12 h, 24 h and 36 h respectively in a 5% CO_2_ atmosphere, the noninvading cells and Matrigel were removed from the upper chamber with a cotton-tipped swab. The inserts were rinsed with PBS, and the cells on the filters were fixed with methanol for 30 min and stained with crystal violet solution (Sigma). The number of invading cells on the filters was counted in five randomly selected fields per filter at 100× magnification. Each experiment was conducted in triplicate.

### Apoptosis assay

Apoptosis-mediated cell death was examined using an APC-Annexin V apoptosis detection kit (BD Biosciences, San Diego, CA, USA) according to the manufacturer’s instructions. Briefly, cells from the LV-B7-H6-shRNA and LV-NC groups were harvested, washed with PBS and resuspended in binding buffer at a density of 1 × 10^6^ cells/mL. Subsequently, 100 µL of the cell suspension was incubated with 5 μL of APC-annexin V solution and 5 μL of propidium iodide (PI) solution. After a 15-min incubation in the dark, the stained cells were analyzed by flow cytometry (BD FACSCanto II, BD Biosciences, NJ, USA), and the data were analyzed using FlowJo 10.0.6 software.

### Cell cycle analysis

Briefly, 1 × 10^6^ cells from the LV-B7-H6-shRNA and LV-NC groups were washed with PBS and were then fixed with 70% ice-cold ethanol at 4 °C overnight. After cells were washed twice with PBS, they were then stained with a solution containing 200 μg/mL PI, 0.1% sodium azide, 0.1% Triton X-100 and 10 μg/mL RNAses for 2–4 h in the dark at room temperature. The stained cells were analyzed for subG1, S and G2 phase peaks by flow cytometry (BD FACSCanto II, BD Biosciences, NJ, USA) using ModFit software.

### Western blot analysis

Cells were homogenized briefly in 10 volumes of lysis buffer containing (in mM) 20 Tris–HCl (pH 7.4), 150 NaCl, 2.5 EDTA, 50 NaF, 0.1 Na_4_P_2_O_7_, 1 Na_3_VO_4_, 1 PMSF, 1 DTT, 0.02% (v/v) protease cocktail (Sigma Aldrich, St. Louis, MO, USA), 1% (v/v) Triton X-100 and 10% (v/v) glycerol. The homogenates were centrifuged twice at 20,000×*g* at 4 °C for 15 min, and the supernatants were retained as total protein. Protein concentrations were determined by the BCA method. Equal amounts of protein were separated by SDS-PAGE and transferred to a PVDF membrane (Merck Millipore, MA, USA). Western blot analysis was performed under standard conditions with specific anti-B7-H6 (1:2000; Abcam, MA, USA), anti-C-myc (1:1500, Abcam, MA, USA), anti-C-fos (1:2000, Cell Signaling Technology, MA, USA), anti-cyclin D1 (1:2000, Cell Signaling Technology, MA, USA), and anti-GAPDH (1:4000, Sigma, St. Louis, MO, USA) antibodies and HRP-labeled goat anti-mouse/rabbit secondary antibody (1:6000, Sigma Aldrich, St. Louis, MO, USA). The immunoreaction was visualized using an enhanced chemiluminescence detection kit (Thermo Fisher, MA, USA) and exposure to X-ray film, and band densities were quantified by densitometry with a video documentation system (Gel Doc 2000, Bio-Rad).

### Statistical analyses

Statistical analysis was conducted by the GraphPad Prism 5.0 software package (GraphPad Software, Inc., San Diego, USA) using a paired Student’s *t*-test, Wilcoxon signed-rank test or Chi square test where appropriate for final analysis of the data. A *P* value < 0.05 was considered statistically significant.

## Results

### B7-H6 expression in human HCC tissues and its correlation with clinical parameters of patients

**Fig. 1:**
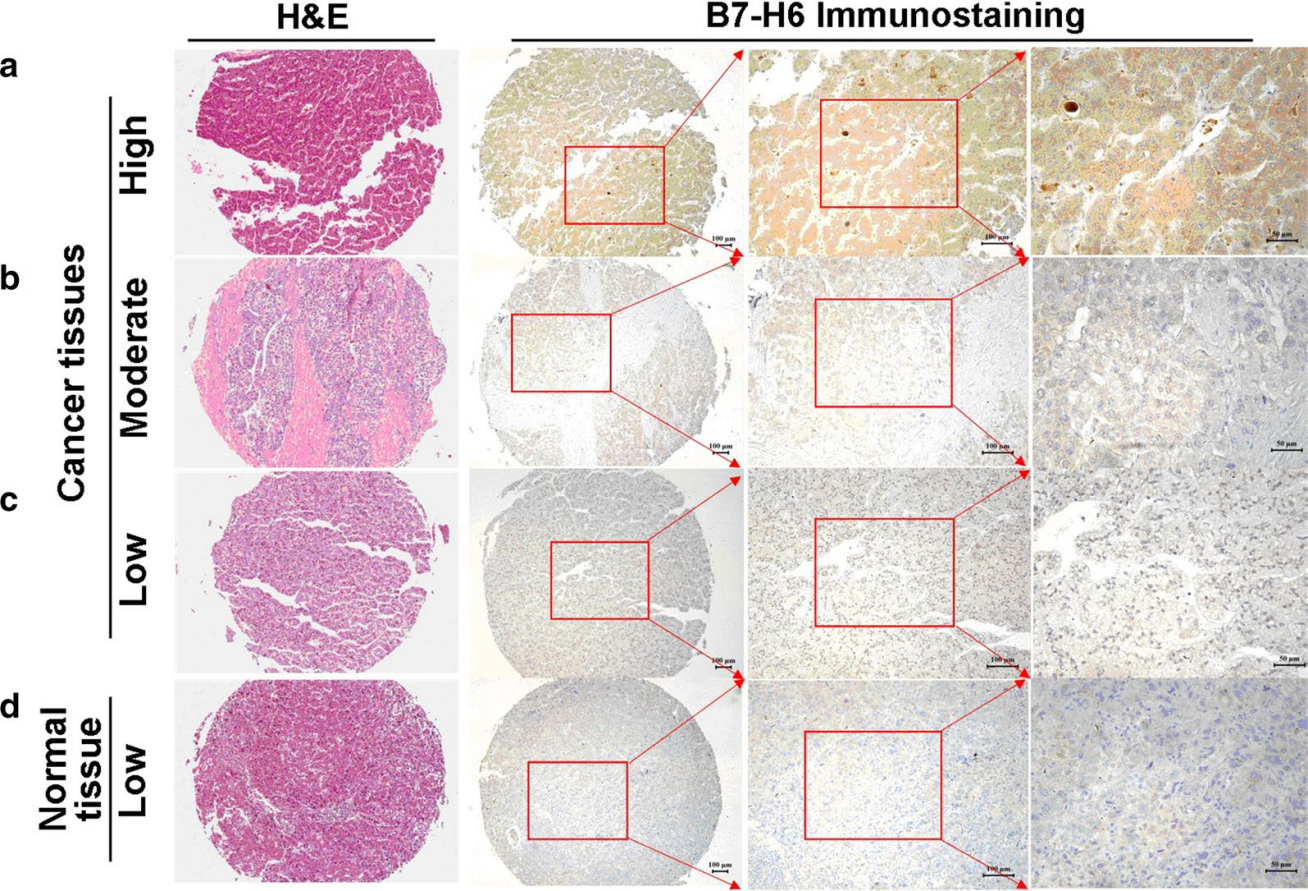
Immunohistochemical staining of B7-H6 in human HCC tissues. Immunohistochemical staining was used to detect B7-H6 expression in human HCC tissues and adjacent normal tissues. Positive B7-H6 staining could be found in the cytoplasm of the cancer cells. **a** High B7-H6 expression in human HCC tissues. **b** Moderate B7-H6 expression in human HCC tissues. **c** Low B7-H6 expression in human HCC tissues. **d** Low B7-H6 expression in adjacent normal tissues. A scale bar = 100 μm or a scale bar = 50 μm was used when needed

**Fig. 2:**
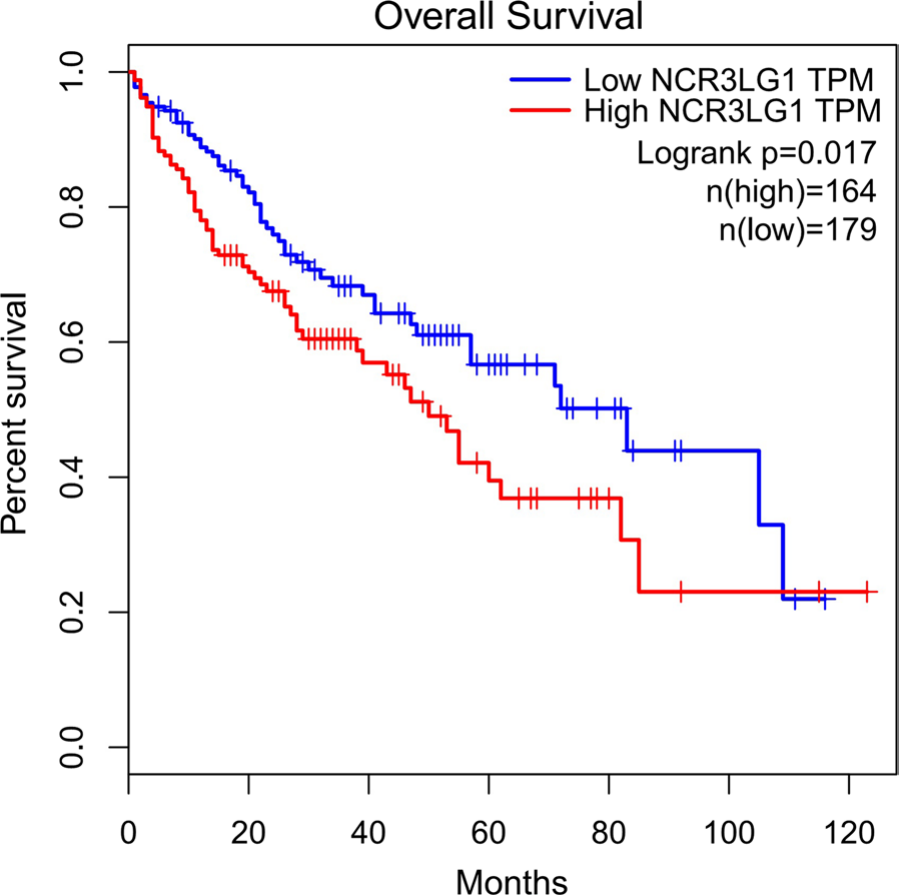
Prognostic value of B7-H6 expression at the mRNA level based on TCGA data. We verified the prognostic value of B7-H6 expression at the mRNA level according to TCGA data from http://gepia.cancer-pku.cn/, and the result showed that lower expression of B7-H6 expression at the mRNA level was significantly associated with better survival in HCC patients (*P *= 0.017)

Immunohistochemical staining was used to examine the B7-H6 expression in both HCC tissues and normal liver tissues. Figure 1 shows that the positive staining for B7-H6 was predominantly localized on the membrane and in the cytoplasm of HCC cells. Figure 1a shows high expression of B7-H6 in HCC tissue. Figure 1b indicates moderate expression of B7-H6 in HCC tissue. Figure 1c represents low expression of B7-H6 in HCC tissue. Figure 1d reveals that weak to moderate staining of B7-H6 could also be found in normal liver tissues. Table 1 summarizes the correlation between the patients’ clinical parameters and B7-H6 expression in the human HCC tissues. Our data demonstrated that B7-H6 expression in the human HCC tissues was significantly associated with the age (*P *= 0.015) and tumor size (*P *= 0.034) of the patients. We did not find any correlation between B7-H6 expression and the other clinical parameters of the patients. Therefore, our data suggested that the overexpression of B7-H6 was involved in the progression of human HCC. Moreover, we also verified the prognostic value of B7-H6 expression at the mRNA level according to TCGA data from http://gepia.cancer-pku.cn/; Fig. 2 shows that lower expression of B7-H6 at the mRNA level was significantly associated with better survival in the HCC patients (*P *= 0.017).

### Knockdown of B7-H6 expression in the HCC cell lines HepG2 and SMMC-7721

**Fig. 3:**
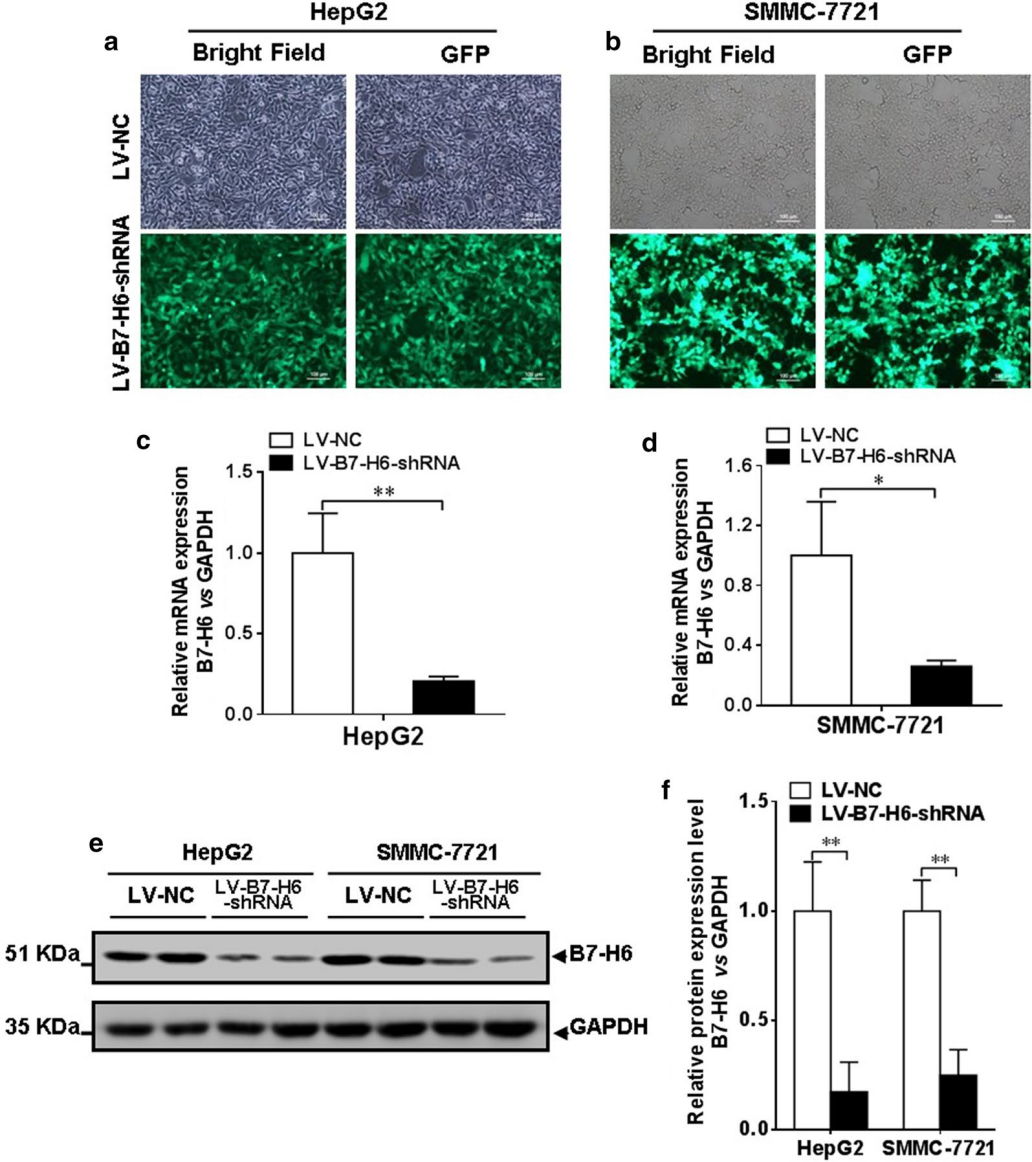
Confirmation of B7-H6 knockdown efficiency in the HCC cell lines HepG2 and SMMC-7721. **a**, **b** Confirmation of the efficiency of infection of the HCC cell lines HepG2 and SMMC-7721 with recombinant lentiviral vector harboring B7-H6 shRNA by the analysis of GFP expression using fluorescence microscopy. **c**, **d** Validation of the decreased B7-H6 expression at the mRNA level after knockdown in HepG2 and SMMC-7721 cells using real-time RT-PCR analysis (*P *< 0.01 and *P *< 0.05, respectively). **e** Detection of B7-H6 expression at the protein level in the LV-NC and LV-B7-H6-shRNA groups of both HepG2 and SMMC-7721 cells using Western blotting. **f** Statistical analysis showed that B7-H6 expression at the protein level was significantly decreased in the LV-B7-H6-shRNA group compared with that in the LV-NC group in both HepG2 and SMMC-7721 cells (both *P *< 0.01)

In the present study, we used the human HCC cell lines HepG2 and SMMC-7721 to assess the role of B7-H6 in the regulation of cellular functions. The knockdown of B7-H6 expression was achieved in both cell lines using shRNA via lentiviral infection, and we then sorted the cells based on GFP expression using a flow sorter. The infection efficiency was also confirmed by detecting GFP expression using fluorescence microscopy (Fig. 3a, b). In addition, we confirmed the knockdown efficiency by real-time RT-PCR analysis, and the present data showed that B7-H6 expression at the mRNA level was significantly decreased in the LV-B7-H6-shRNA group compared with that in the LV-NC group in both the HepG2 and SMMC-7721 cell lines (*P *< 0.01 and *P *< 0.05 for HepG2 and SMMC-7721, respectively; Fig. 3c, d). In addition, Western blot analysis confirmed that the expression of B7-H6 at the protein level in the LV-B7-H6-shRNA group was decreased compared with that in the LV-NC group in both HCC cell lines (Fig. 3e, f, both *P *< 0.01).

### Knockdown of B7-H6 expression significantly suppresses proliferation, migration, and invasion and induces cell cycle arrest in human HCC cell lines

**Fig. 4:**
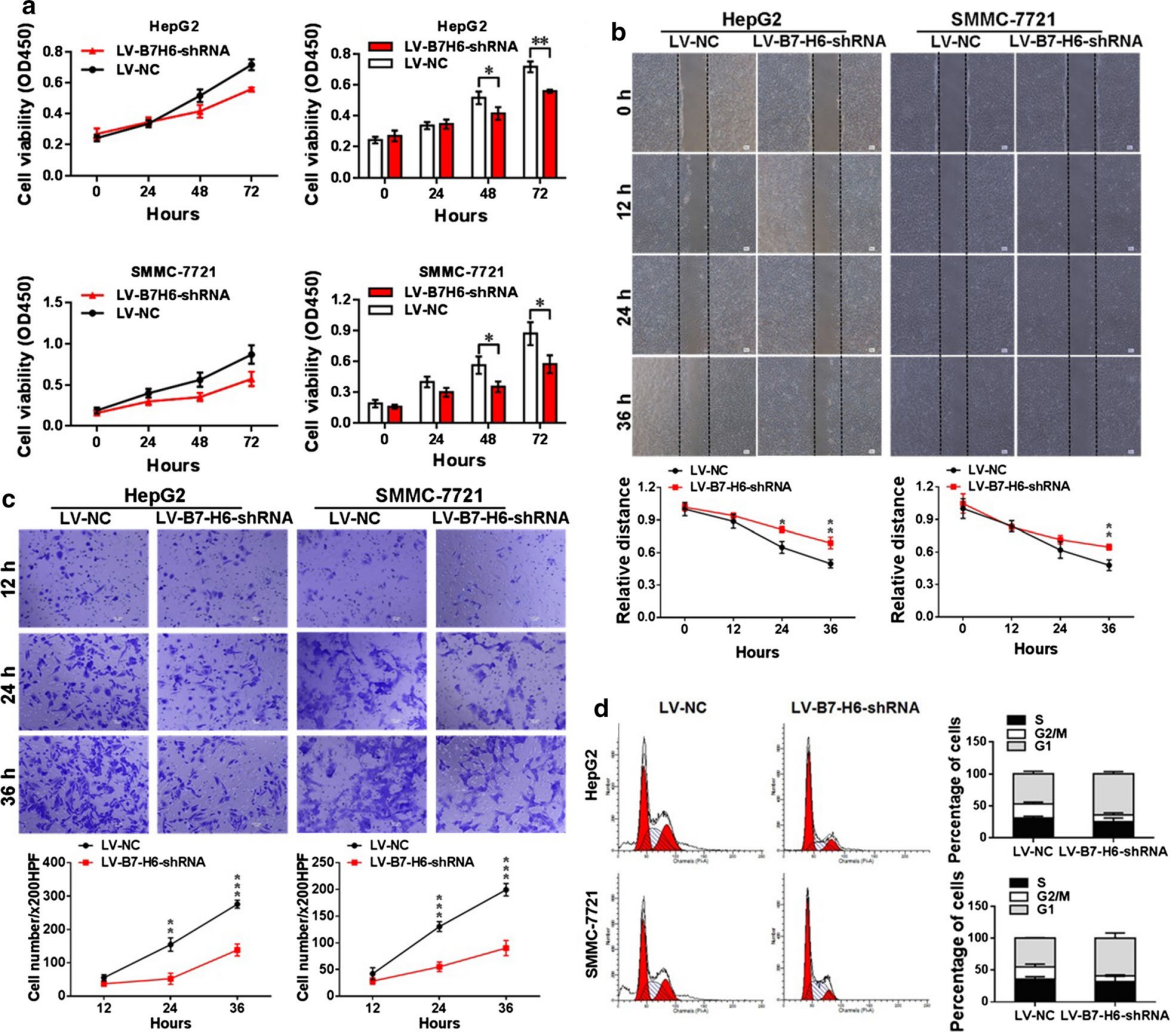
Contribution of B7-H6 knockdown to cell proliferation, migration, invasion and cell cycle in HCC cell lines. **a** The CCK-8 assay was used to examine the effects of B7-H6 knockdown on the cell proliferation rate in vitro in the human HCC cell lines HepG2 and SMMC-7721. In HepG2 cells, the proliferation rate of the LV-B7-H6-shRNA group was significantly lower than that of the LV-NC group at 48 h and 72 h (*P *< 0.05 and *P *< 0.01, respectively). In SMMC-7721 cells, the proliferation rate of the LV-B7-H6-shRNA group was significantly lower than that of the LV-NC group at 48 h and 72 h (both *P *< 0.05). **b** A wound healing assay was performed to examine the contribution of B7-H6 knockdown to the migration of HCC cells. In HepG2 cells, the cell-free area of the LV-B7-H6-shRNA group was significantly wider than that of the LV-NC group at 24 h and 36 h (*P *< 0.05 and *P *< 0.01 respectively) after the scratch was made on the cell monolayer, and in SMMC-7721 cells, the cell-free area of the LV-B7-H6-shRNA group was significantly wider than that of the LV-NC group at 36 h (*P *< 0.01) after the scratch was made on the cell monolayer. **c** The Transwell invasion assay indicated that in both HepG2 and SMMC-7721 cells, the number of crystal violet-stained cells was significantly decreased in the LV-B7-H6-shRNA group compared with that in the LV-NC group (at 24 h, *P *< 0.01 and *P *< 0.001 respectively, and at 36 h, both *P *< 0.001). **d** The flow cytometric analysis showed that in both HepG2 and SMMC-7721 cells, the LV-B7-H6-shRNA group displayed an increased percentage of cells in the G1 phase and a decreased percentage of cells in the G2/M phases compared with the LV-NC group

We assessed the cell proliferation rate of the human HCC cell lines HepG2 and SMMC-7721 after knockdown of B7-H6 expression in both the LV-B7-H6-shRNA and LV-NC groups using a CCK-8 assay. Figure 4a shows that in HepG2 cells, the proliferation rate of the LV-B7-H6-shRNA group was significantly lower than that of the LV-NC group after 48 and 72 h (*P *< 0.05 and *P *< 0.01, respectively), and in SMMC-7721 cells, the proliferation rate of the LV-B7-H6-shRNA group was significantly lower than that of the LV-NC group after 48 and 72 h (both *P *< 0.05). Moreover, Fig. 4b shows that in the wound healing assay, in HepG2 cells, the cell-free area of the LV-B7-H6-shRNA group was significantly wider than that of the LV-NC group at 24 h and 36 h (*P *< 0.05 and *P *< 0.01 respectively) after the scratch was made on the cell monolayer, and in SMMC-7721 cells, the cell-free area of the LV-B7-H6-shRNA group was significantly wider than that of the LV-NC group at 36 h (*P *< 0.01) after the scratch was made on the cell monolayer. The Transwell invasion assay indicated that in both HepG2 and SMMC-7721 cells, the number of crystal violet-stained cells was significantly decreased in the LV-B7-H6-shRNA group compared with that in the LV-NC group (at 24 h, *P *< 0.01 and *P *< 0.001 respectively, and at 36 h, both *P *< 0.001) (Fig. 4c). We further tested the contribution of B7-H6 expression to HCC cell apoptosis and cell cycle regulation. Figure 4d shows that in both HepG2 and SMMC-7721 cells, the LV-B7-H6-shRNA group displayed an increased percentage of cells in the G1 phase and a decreased percentage in the G2/M phases compared with the LV-NC group. Therefore, our present data supported the notion that the knockdown of B7-H6 expression could induce cell cycle arrest in HCC cells.

### B7-H6 knockdown significantly decreases the expression of C-myc, C-fos and cyclin D1 in HCC cell lines

**Fig. 5:**
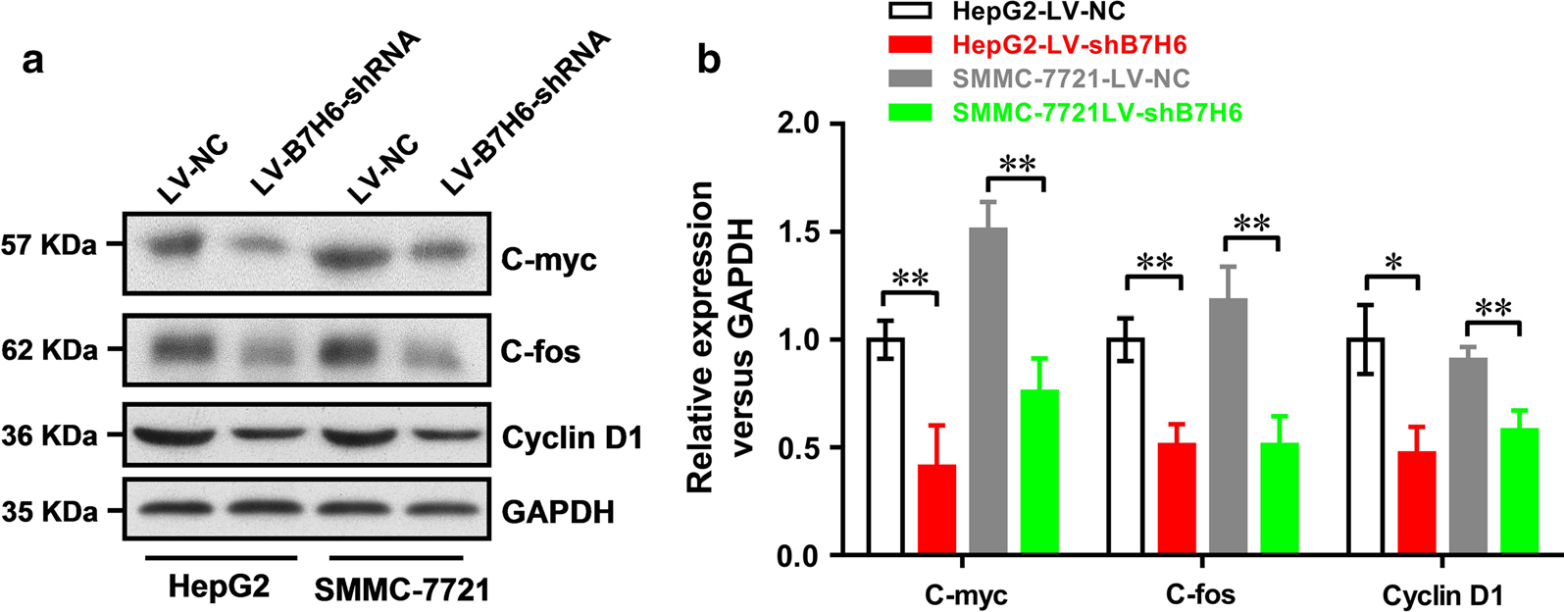
B7-H6 knockdown significantly decreased the expression of C-myc, C-fos and cyclin D1 in HCC cell lines. **a** Western blotting was performed to examine the protein expression levels of C-myc, C-fos and cyclin D1, which are involved in the regulation of cell proliferation and the cell cycle. **b**. Statistical analysis showed that in both HepG2 and SMMC-7721 cells, B7-H6 knockdown could significantly decrease the expression of C-myc (both *P *< 0.01), C-fos (both *P *< 0.01) and cyclin D1 (*P *< 0.05 and *P *< 0.01, respectively)

Because the knockdown of B7-H6 expression significantly suppresses proliferation and induces cell cycle arrest in human HCC cell lines, we next carried out Western blotting to examine the protein expression levels of C-myc, C-fos and cyclin D1, which are involved in the regulation of cell proliferation and the cell cycle. As shown in Fig. 5a, b, in both HepG2 and SMMC-7721 cells, B7-H6 knockdown could significantly decrease the expression of C-myc (both *P *< 0.01), C-fos (both *P *< 0.01) and cyclin D1 (*P *< 0.05 and *P *< 0.01, respectively), which further confirmed that B7-H6 was involved in the regulation of cell proliferation and the cell cycle.

## Discussion

It has been widely accepted that chronic inflammation plays an important role in the tumor microenvironment and that it is a critical cause of oncogenesis and cancer progression; thus, chronic inflammation is regarded as a hallmark of cancer [19]. Up to 80% of the HCC cases in China are attributable to HBV infection [20]. Li et al. have demonstrated that chronic HBV infection can hinder NK cell function in patients with chronic hepatitis B, and they provided evidence of veritable NK cell immunity during different natural history phases in treatment-naïve patients with chronic HBV infection [21]. Moreover, NK cell-mediated tumor cell eradication contributes importantly to the oncogenesis and progression of human HCC [22]. It has been demonstrated that NKp30-B7-H6 interaction can aggravate hepatocyte damage through up-regulation of interleukin-32 in HBV-related acute-on-chronic liver failure [11]. However, up to now, how B7-H6 influences the cancer progression of HCC has still remained largely unexplored.

As a newly discovered member of the B7 family, B7-H6 interacts with its receptor, namely, NKp30, on NK cells and plays an important role in NK cell-mediated immune responses [2]. Abnormal B7-H6 expression can be found in many human cancer tissues, suggesting its important clinical significance. We have previously reported that higher B7-H6 expression in ovarian cancer tissues is positively correlated with tumor metastasis and cancer progression [8]. Jiang et al. have also shown that the knockdown of B7-H6 expression in U87 and U251 glioma cells significantly suppresses cell proliferation, migration and invasion, leading to enhanced apoptosis along with the induction of cell cycle arrest [5]. Moreover, Che et al. also confirmed the contribution of B7-H6 in cellular function regulation of human glioma cells, and moreover, they also showed that, the knockdown of B7-H6 could also induce the increased expression levels of E-cadherin and Bcl-2 associated X protein, and the decreased expression levels of vimentin, N-cadherin, MMP-2, MMP-9 and survivin, which further supported that B7-H6 was involved in the promotion of cancer progression [23]. Wu et al. have also demonstrated that B7-H6 is widely expressed in B-cell lymphomas and that the knockdown of B7-H6 expression can not only inhibit the proliferation, colony formation, migration and invasion of lymphoma cells but can also increase cell apoptosis and sensitivity to vincristine and dexamethasone [24]. All these data support the ideas that B7-H6 expression is involved in the progression of human cancers and that B7-H6 plays an important role in regulating the biological behavior of cancer cells. Therefore, B7-H6 is a potential clinical biomarker for several human cancers.

In the present study, we identified the expression pattern of B7-H6 in human HCC tissues and found that the B7-H6 expression level in HCC tissues was significantly associated with tumor size. Furthermore, TCGA data also showed that lower expression of B7-H6 at the mRNA level was significantly associated with better survival in HCC patients. Then, we successfully constructed a cellular model for the knockdown of B7-H6 expression in human HCC cell lines using the RNAi method, and the contribution of B7-H6 to various cellular features of HCC was assessed. Our data demonstrated that the knockdown of B7-H6 expression in the HCC cell lines HepG2 and SMMC-7721 could significantly suppress cell proliferation, migration and invasion and could lead to cell cycle arrest at the G1 phase, indicating that B7-H6 was involved in regulating the biological behavior of HCC cells. Moreover, we found that B7-H6 knockdown could significantly decrease the expression of C-myc, C-fos and cyclin D1 in HCC cell lines, which further confirmed that B7-H6 was involved in the regulation of cell proliferation and the cell cycle. Recently, Wu et al. have reported that B7-H6-specific chimeric antigen receptors lead to tumor elimination and enhanced host antitumor immunity [25]. Collectively, all these data suggested that B7-H6 could be used for the development of immunotherapeutic approaches targeting human HCC.

## Conclusions

Our present findings suggested that B7-H6 played an important role in regulating the biological behavior of HCC cells and that B7-H6 could be used for the development of immunotherapeutic approaches targeting this malignancy.

## Abbreviations

NK: natural killer
HCC: hepatocellular carcinoma
HBV: hepatitis B virus
H&E: hematoxylin and eosin
FBS: fetal bovine serum
DMEM: Dulbecco’s modified Eagle’s medium
PBS: phosphate-buffered saline
shRNA: small hairpin RNA
GFP: green fluorescent protein
CCK-8: cell counting kit-8
PI: propidium iodide
