# Correction to: Expression of B7-H6 expression in human hepatocellular carcinoma and its clinical significance

**DOI:** 10.1186/s12935-018-0636-6

**Published:** 2018-09-10

**Authors:** Lujun Chen, Jun Feng, Bin Xu, You Zhou, Xiao Zheng, Changping Wu, Jingting Jiang

**Affiliations:** 1 Department of Tumor Biological Treatment and Research Center for Cancer Immunotherapy Technology of Jiangsu Province, The Third Affiliated Hospital of Soochow University, Changzhou, 213003 Jiangsu China; 2 Jiangsu Engineering Research Center for Tumor Immunotherapy, The Third Affiliated Hospital of Soochow University, Changzhou, 213003 Jiangsu China; 3 Institute of Cell Therapy, The Third Affiliated Hospital of Soochow University, Changzhou, 213003 Jiangsu China

## Correction to: Cancer Cell Int (2018) 18:126 10.1186/s12935-018-0627-7

Following publication of the original article [1], the authors reported a typing mistake in the title. In this Correction the incorrect and correct titles are shown.

Incorrect title:

- Expression of B7-H6 expression in human hepatocellular carcinoma and its clinical significance

The correct title is:

- B7-H6 expression in human hepatocellular carcinoma and its clinical significance

## References

[1] Chen L, Feng J, Xu B, Zhou Y, Zheng X, Wu C, Jiang J (2018). Expression of B7-H6 expression in human hepatocellular carcinoma and its clinical significance. Cancer Cell Int.

